# Performance comparison of two nucleic acid amplification systems for SARS‐CoV‐2 detection: A multi‐center study

**DOI:** 10.1002/jcla.24727

**Published:** 2022-10-04

**Authors:** Chan Mo, Kamfai Lo, Ying He, Bo Peng, Feifan Guo, Zhou Zheng, Ruiwei Jiang, Yihua Cai, Yumin Li, Dongyue Guo, Bing Zhang, Tong Ou, Dan Xiong, Xiuming Zhang

**Affiliations:** ^1^ Medical Laboratory of the Third Affiliated Hospital of Shenzhen University Shenzhen Guangdong China; ^2^ The University of Hong Kong‐Shenzhen hospital Shenzhen Guangdong China; ^3^ The Eighth Affiliated Hospital of Sun Yat‐sen University Shenzhen Guangdong China; ^4^ Shenzhen Center for Disease Control and Prevention Shenzhen Guangdong China; ^5^ Anhui University of Science and Technology Huainan Anhui China

**Keywords:** clinical detection performance, multi‐center study, nucleic acid detection system, SARS‐CoV‐2

## Abstract

**Background:**

Many rapid nucleic acid testing systems have emerged to halt the development and spread of COVID‐19. However, so far relatively few studies have compared the diagnostic performance between these testing systems and conventional detection systems. Here, we performed a retrospective analysis to evaluate the clinical detection performance between SARS‐CoV‐2 rapid and conventional nucleic acid detection system.

**Methods:**

Clinical detection results of 63,352 oropharyngeal swabs by both systems were finally enrolled in this analysis. Sensitivity (SE), specificity (SP), and positive and negative predictive value (PPV, NPV) of both systems were calculated to evaluate their diagnostic accuracy. Concordance between these two systems were assessed by overall, positive, negative percent agreement (OPA, PPA, NPA) and κ value. Sensitivity of SARS‐CoV‐2 rapid nucleic acid detection system (Daan Gene) was further analyzed with respect to the viral load of clinical specimens.

**Results:**

Sensitivity of Daan Gene was slightly lower than that of conventional detection system (0.86 vs. 0.979), but their specificity was equivalent. Daan Gene had ≥98.0% PPV and NPV for SARS‐CoV‐2. Moreover, Daan Gene demonstrated an excellent test agreement with conventional detection system (κ = 0.893, *p* = 0.000). Daan Gene was 99.31% sensitivity for specimens with high viral load (*C*_t_ < 35) and 50% for low viral load (*C*_t_ ≥ 35).

**Conclusions:**

While showing an analytical sensitivity slightly below than that of conventional detection system, rapid nucleic acid detection system may be a diagnostic alternative to rapidly identify SARS‐CoV‐2‐infected individuals with high viral loads and a powerful complement to current detection methods.

## Graphical abstract

Many rapid nucleic acid testing systems have emerged to halt the development and spread of COVID‐19. However, so far relatively few studies have compared the diagnostic performance between these testing systems and conventional detection systems. Here, we performed a retrospective analysis to evaluate the clinical detection performance between SARS‐CoV‐2 rapid and conventional nucleic acid detection system. While showing an analytical sensitivity slightly below than that of conventional detection system, rapid nucleic acid testing systems may be a diagnostic alternative to rapidly identify SARS‐CoV‐2 infected individuals with high viral loads and a powerful complement to current detection methods.

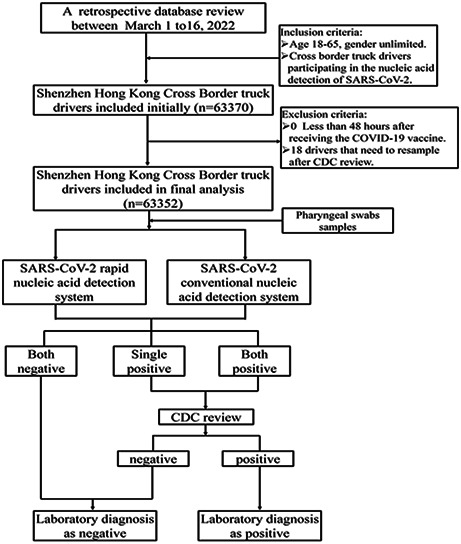

## 1 INTRODUCTION

COVID‐19 caused by SARS‐CoV‐2 is a new infectious disease following SARS and MERS. Globally, as of 5:54 pm CEST, September 6, 2022, there have been 603,164,436 confirmed cases of COVID‐19, including 6,482,338 deaths, reported to WHO (https://covid19.who.int/). Rapid spread of SARS‐CoV‐2 and its variants had brought a huge burden to the global economy and medical system.^1^, ^2^ Early diagnosis, supportive care, isolation of infected patients, and their contacts are key to epidemic prevention and control. Rapid, sensitive, and inexpensive methods to detect SARS‐CoV‐2 infection are therefore urgently needed and ultimately to halt the development and spread of COVID‐19. Conventional nucleic acid detection system is highly sensitive and specific (though not 100% for each), rapid and widely used for pathogen detection and is currently the main method for diagnosing COVID‐19.^3^ On the other hand, it has disadvantages, such as site restrictions for specialty laboratory zones, highly dependent on thermal circulator, multiple detection steps and long time consuming, which makes it difficult to meet the increasing needs of nucleic acid screening and rapid detection in outpatient and emergency departments.^4^, ^5^, ^6^, ^7^ In order to meet this challenge, many rapid nucleic acid detection systems for SARS‐CoV‐2 have been born one after another, the whole process of their detection time were significantly shortened, and the instrument miniaturization (part of the detection system are portable), with or without the characteristics of nucleic acid extraction, amplification and detection integration, as contrast to conventional SARS‐CoV‐2 nucleic acid detection systems. Since these methods used now have not met the requirements for point‐of‐care testing (POCT), they were temporarily named as “rapid nucleic acid testing” in *Chinese expert consensus on the rapid nucleic acid testing of SARS‐CoV‐2*. The analytical and clinical performance of these newly rapid nucleic acid testing for SARS‐CoV‐2 have been rarely evaluated based on the Chinese population thus far. Even if there are similar studies, they generally have some weaknesses, such as small scale of clinical validation trials and insufficient scientific data support.^8^, ^9^, ^10^, ^11^, ^12^ Therefore, the objective of present study was to evaluate the clinical diagnostic performance of SARS‐CoV‐2 rapid nucleic acid detection system (Daan Gene) compared with SARS‐CoV‐2 conventional nucleic acid detection system in a large cohort of oropharyngeal swabs samples.

## 2 MATERIALS AND METHODS

### 2.1 Study subjects

**FIGURE 1:**
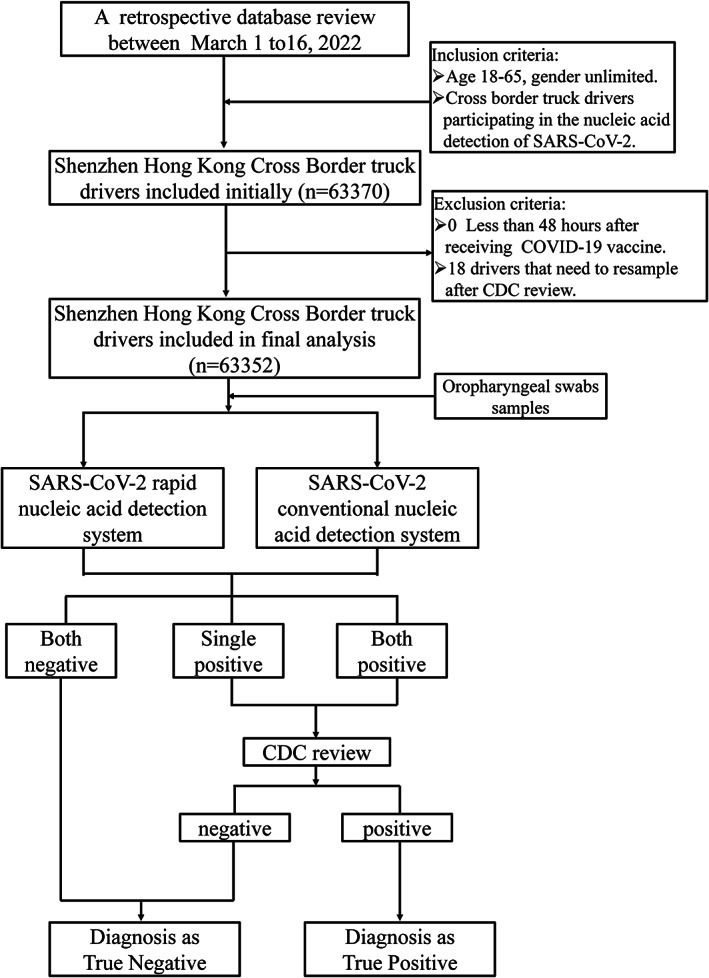
Study design and selection of clinical data

Oropharyngeal swabs specimens, which were obtained for rapid and conventional nucleic acid detection, collected from a total of 63,370 Shenzhen Hong Kong Cross Border truck drivers at six SARS‐CoV‐2 nucleic acid testing laboratories in Shenzhen China from March 1, to 16, 2022. The ages of the drivers enrolled in this study were between 18 and 65 years and gender unlimited. Exclusion criteria were as follows: <48 h after receiving SARS‐CoV‐2 vaccine. Samples that needed to resample after CDC re‐tested (*n* = 18). After these exclusions, 63,352 cases of Shenzhen Hong Kong Cross Border truck drivers' nucleic acid test results were finally included in the analysis. Nucleic acid test results of both systems (including reported results of negative specimens, cycle threshold [*C*_t_] values of suspected positive and positive specimens) and reported results after CDC review were collected from laboratory information management system records. The study design is displayed in Figure 1.

### 2.2 Clinical specimens detected by SARS‐CoV‐2 rapid nucleic acid detection system (Daan Gene)

Nucleic acid extraction was performed after oropharyngeal swabs specimens were collected from truck drivers according to the “Technical Guidelines for Laboratory Testing of Pneumonia Infected with SARS‐CoV‐2” issued by the National Health Commission of the people's Republic of China. Then, rapid nucleic acid detection was performed on AGS8830 instrument according to the instructions of the SARS‐CoV‐2 nucleic acid detection Kit (Daan Gene Co., Ltd). Briefly, the supporting kit is a single tube and single copy packaging specification, and there is no need to prepare the reaction system. The reagent can be transferred to the amplification detection area after instantaneous centrifugation for 15 s, shaking and mixing for 10 s, and then instantaneous centrifugation for 15 s. Amplification procedure: 50°C 2 min → 95°C 2 min → (95°C 5 s, 60°C 10 s 10 cycles) → (95°C 5 s, 60°C 10 s 32 cycles).

### 2.3 Clinical specimens detected by SARS‐CoV‐2 conventional nucleic acid detection system

The process of specimen collection and nucleic acid extraction was the same as described in Section 2.2. In Shatoujiao Port (STJP), Liantang port (LTP), Wenjindu port (WJDP) Medical Laboratory of Shenzhen Luohu hospital group and Mobile PCR laboratory of Shenzhen Hospital of the University of Hong Kong (Shenzhen Bay Port, SZBP), conventional nucleic acid detection was performed on ABI7500 fluorescence quantitative PCR instrument according to the instructions of the SARS‐CoV‐2 nucleic acid detection Kit (Biogerm Medical Technology Co., Ltd.). Add 5 μl nucleic acid of the tested sample, positive control, and negative control into the prepared PCR reaction tube, respectively, with a final volume of 25 μl/tube. PCR amplification detection can be carried out after instantaneous centrifugation. Cycle parameters were set as follows: 50°C for 10 min →95°C for 5 min → (95°C for 10 s, 55°C for 40 s 45 cycles).

In Huanggang port laboratory (HGP) and Channel 1 laboratory of Futian Free Trade Zone (FTFTZ) of the Eighth Affiliated Hospital of Sun Yat‐sen University, conventional nucleic acid detection was carried out on ABI7500 fluorescence quantitative PCR instrument followed the instructions of the SARS‐CoV‐2 nucleic acid detection Kit (Sansure Biotech Inc.). PCR amplification cycle parameters were set as follows: 50°C for 30 min →95°C for 1 min → (95°C for 15 s, 60°C for 30 s 45 cycles).

### 2.4 Suspected positive and positive specimens re‐tested by Shenzhen Center for Disease Control and Prevention (CDC)

For the specimens suspected to be positive or positive in the initial test of port laboratory, conventional nucleic acid detection was carried out using both SARS‐CoV‐2 nucleic acid detection kits (Biogerm Medical Technology Co., Ltd. and Daan Gene Co., Ltd.) to re‐test the specimens by Shenzhen CDC. According to *the Technical Guidelines for COVID‐19 Laboratory Testing (Ninth Edition)* issued by China CDC, the case confirmed as positive in the laboratory was further sequenced to monitor the mutation of virus genome, provide experimental data for the change of COVID‐19 nucleic acid detection reagent, vaccine research and development strategy, and also provide support for the work of epidemiological control and traceability.

### 2.5 Diagnostic accuracy criteria

True positive cases in this study were confirmed by Shenzhen CDC in reference to *the Technical Guidelines for COVID‐19 Laboratory Testing (Ninth Edition)* issued by China CDC^13^: One of the following conditions was met: (a): both of the SARS‐CoV‐2 targets (ORF1ab, N) in the same specimen were positive by real‐time RT‐PCR. When a single target was positive, retesting or re‐sampling was required. (b): When a single target was positive in real‐time RT‐PCR of two specimens at the same time, or when a single target was positive in two sampling tests of the same type of specimens, it was diagnosed as positive.

True negative cases in this study were confirmed in reference to *the Technical Guidelines for COVID‐19 Laboratory Testing (Ninth Edition)* issued by China CDC^13^: Two consecutive SARS‐CoV‐2 nucleic acid tests were negative.

### 2.6 Statistical methods

Analysis was performed according to the *Guideline for evaluation of qualitative test performance* (WS/T505‐2017) issued by National Health and Family Planning Commission of the people's Republic of China and the *Guidance on the Performance Verification for Molecular Diagnostic Procedures* (CNAS‐GL039) issued by China National Accreditation Committee for Conformity Assessment. To evaluate the diagnostic accuracy of these systems, sensitivities (SE), specificities (SP), positive predictive value (PPV), negative predictive value (NPV), and 95% confidence interval (CI) were calculated based on the diagnostic accuracy criteria. CI for proportions were calculated by the Wilson method. Concordance between these two systems was assessed by the overall percent agreement (OPA), positive percent agreement (PPA), negative percent agreement (NPA), and Cohen's kappa value (κ) which was performed using SPSS™ (20.0, IBM Corp) and classified as follows: 0.81–1, almost perfect; 0.61–0.80, substantial; 0.41–0.60, moderate; 0.21–0.40, fair; 0.0–0.20, slight.^14^, ^15^ A *p* value <0.05 was considered to be significant. *C*_t_ values of the inconsistent specimens between the two systems were recorded and presented in the form of *median ± IQR*.

## 3 RESULTS

### 3.1 Comparison of diagnostic performance of SARS‐CoV‐2 rapid nucleic acid detection system (Daan Gene) and SARS‐CoV‐2 conventional nucleic acid detection system

Clinical detection results of 63,352 oropharyngeal swabs by both systems were finally enrolled in this analysis. As described in Table 1, the sensitivity of SARS‐CoV‐2 conventional nucleic acid detection system was higher than that of SARS‐CoV‐2 rapid nucleic acid detection system (Daan Gene) (0.979, 95% CI: 0.949–0.992 vs. 0.860, 95% CI: 0.808–0.901) slightly, and their specificities were equivalent (0.999, 95% CI: 0.999–1.0). The PPV of SARS‐CoV‐2 rapid nucleic acid detection system (Daan Gene) was found to be 0.985(95% CI, 0.955–0.996), and 0.947(95% CI, 0.909–0.970) for SARS‐CoV‐2 conventional nucleic acid detection system. The NPV of both systems for detecting SARS‐CoV‐2 were above 0.999.

**TABLE 1:** Diagnostic accuracy of SARS‐CoV‐2 rapid and conventional nucleic acid detection systems for SARS‐CoV‐2 detection

Test systems	Diagnostic accuracy criteria		SE (95% CI)	SP (95% CI)	PPV (95% CI)	NPV (95% CI)
	Positive	Negative				
Positive^1^	203^a^	3^b^	0.860 (0.808–0.901)	0.999 (0.999–1)	0.985 (0.955–0.996)	0.999 (0.999–1)
Negative^1^	33^c^	63,113^d^				
Positive^2^	231	13	0.979 (0.949–0.992)	0.999 (0.999–1)	0.947 (0.909–0.970)	0.999 (0.999–1)
Negative^2^	5	63,103				

*Note*: SE = a/(a + c); SP = d/(b + d); PPV = a/(a + b); NPV = d/(c + d).

^1^ SARS‐CoV‐2 rapid nucleic acid detection system.

^2^ SARS‐CoV‐2 conventional nucleic acid detection system.

Taken together, our results indicated that both systems exhibited a perfect agreement with diagnostic accuracy criteria, and SARS‐CoV‐2 conventional nucleic acid detection system shown a more preferable agreement.

### 3.2 Concordance between SARS‐CoV‐2 rapid nucleic acid detection system (Daan Gene) and SARS‐CoV‐2 conventional nucleic acid detection system

In general, agreement between these two systems for individual SARS‐CoV‐2 nucleic acid detection were almost perfect (κ = 0.893, *p* = 0.00), with an OPA of 0.999 (95% CI: 0.998–1.0), PPA of 0.824(95% CI: 0.769–0.868), NPA of 0.999 (95% CI: 0.999–1), as reported in Table 2.

**TABLE 2:** Concordance between SARS‐CoV‐2 rapid and conventional nucleic acid detection systems

Test system^1^	Reference system^2^		OPA (95% CI)	PPA (95% CI)	NPA (95% CI)	ĸ	*p* Value
	Positive	Negative					
Positive	201^a^	5^b^	0.999 (0.998–1)	0.824 (0.769–0.868)	0.999 (0.999–1)	0.893	0.000
Negative	43^c^	63,103^d^					

*Note*: OPA = (a + d)/(a + b + c + d); PPA = a/(a + c); NPA = d/(b + d).

^1^ Test system: SARS‐CoV‐2 rapid nucleic acid detection system.

^2^ Reference system: SARS‐CoV‐2 conventional nucleic acid detection system.

### 3.3 Discordant specimens between SARS‐CoV‐2 rapid nucleic acid detection system (Daan Gene) and SARS‐CoV‐2 conventional nucleic acid detection system

**FIGURE 2:**
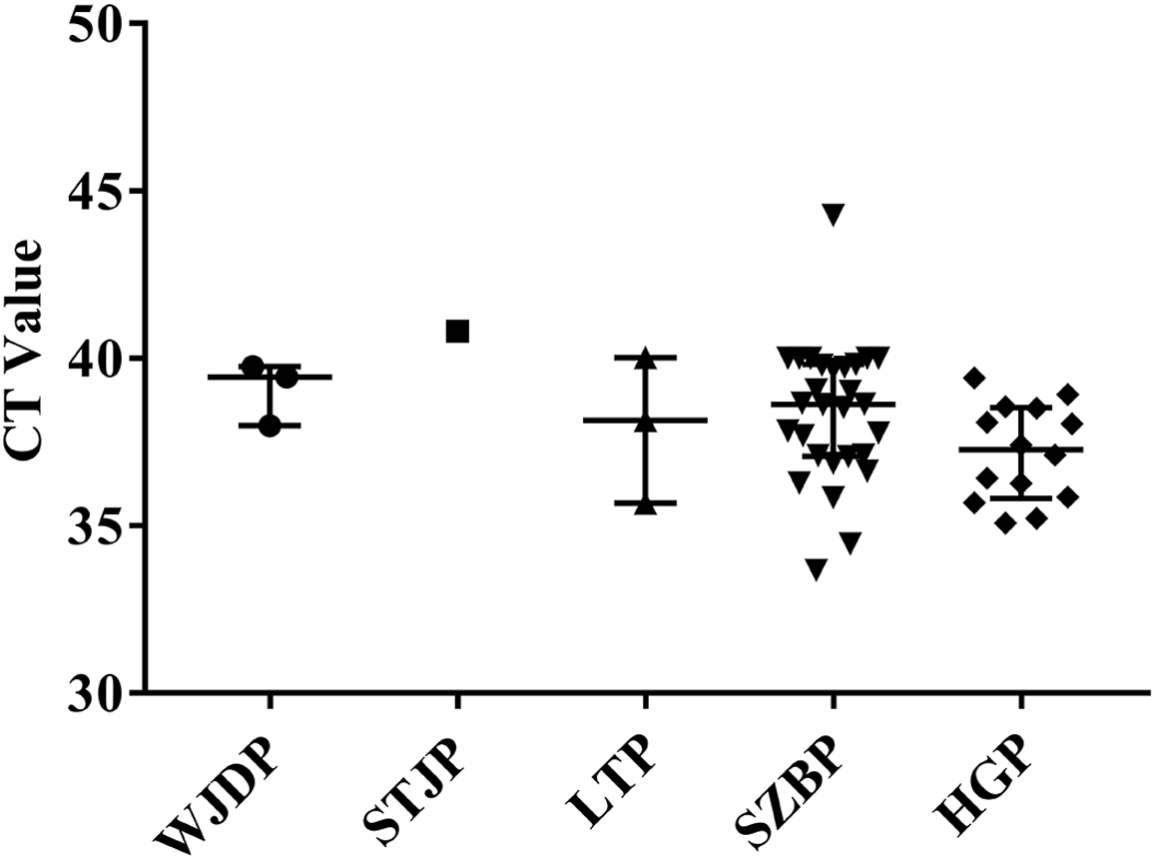
The distribution of Ct values with respect to the discordant specimens between SARS‐CoV‐2 rapid and conventional nucleic acid detection system

The distribution of *C*_t_ values with respect to the discordant specimens between SARS‐CoV‐2 rapid nucleic acid detection system (Daan Gene) and SARS‐CoV‐2 conventional nucleic acid detection system is displayed in Figure 2. Discordant specimens all had relatively high *C*_t_ values (indicative of a lower viral concentration in the specimen), ranged from 33.67 to 44.26, with median of 38.14, IQR of 2.95.

### 3.4 Sensitivity of SARS‐CoV‐2 rapid nucleic acid detection system (Daan Gene) in respect to clinical specimens with different viral load

**FIGURE 3:**
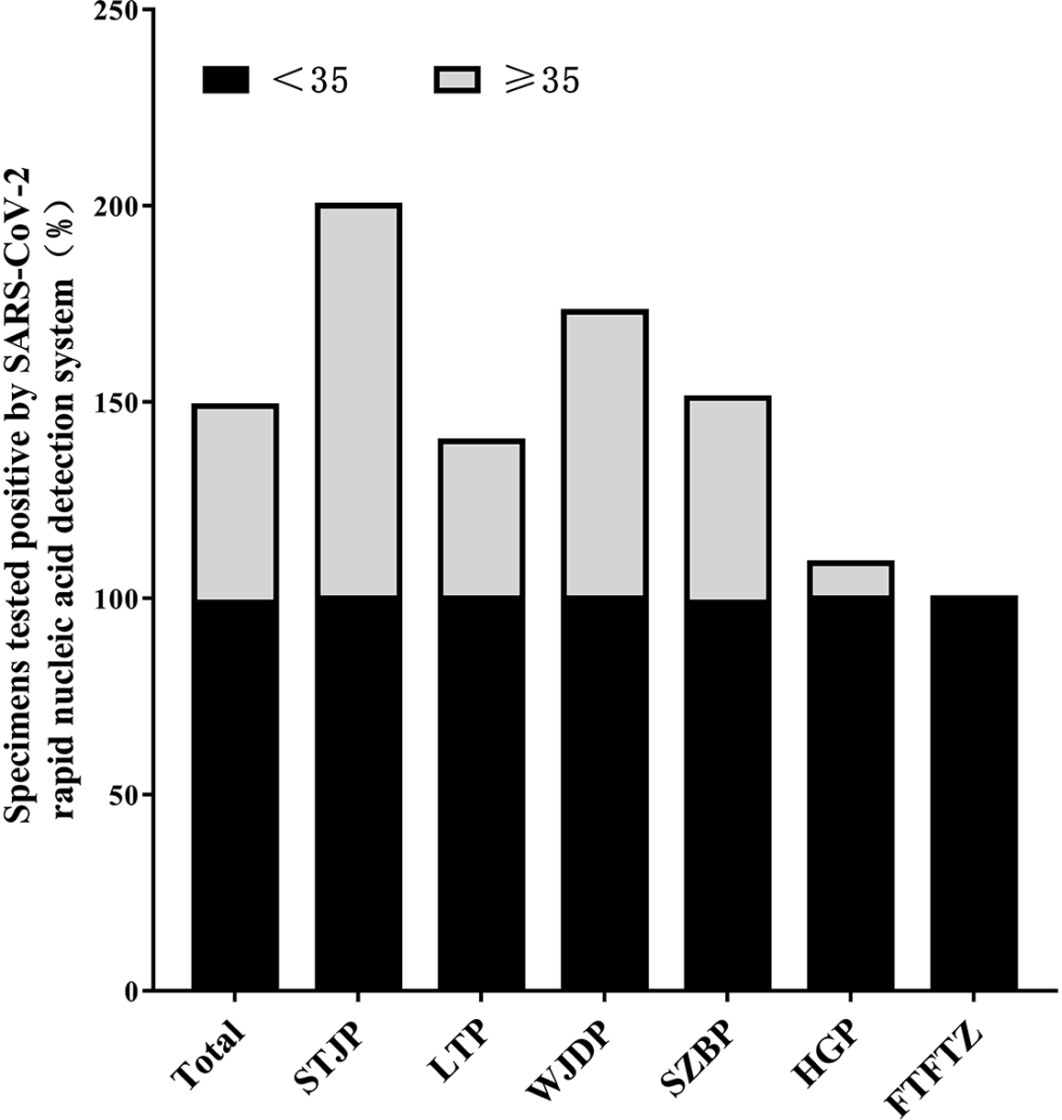
Sensitivity of SARS‐CoV‐2 rapid nucleic acid detection system (Daan Gene) in respect to clinical specimens with different viral load

The diagnostic ability of detection system is closely related to the virus load of the tested sample. We further divided the viral loads of samples by *C*_t_ values and determined the sensitivity of rapid nucleic acid detection system (Daan Gene) under different viral loads (Figure 3). Overall, total test data of six port laboratories shown the sensitivity of SARS‐CoV‐2 rapid nucleic acid detection system (Daan Gene) was 99.31% for samples with a high viral load (*C*_t_ < 35), 50% for low viral load (*C*_t_ ≥ 35). The sensitivity of SARS‐CoV‐2 rapid nucleic acid detection system (Daan Gene) was 100% for specimens with a high viral load (*C*_t_ < 35) in STJP, LTP, WJDP, HGP, and FTFTZ medical laboratories, and 98.89% in SZBP medical laboratory. Moreover, for samples with a low viral load (*C*_t_ ≥ 35), the diagnostic sensitivity of SARS‐CoV‐2 rapid nucleic acid detection system (Daan Gene) was 100%, 40%, 72.72%, 52.38%, and 9.09% in STJP, LTP, WJDP, SZBP, and HGP medical laboratories, respectively.

## 4 DISCUSSION

Etiological examination is an important basis for the diagnosis of SARS‐CoV‐2 infection, including viral nucleic acid and antibody. Virus nucleic acid detection is the earliest and most commonly method applied to confirm the infection of SARS‐CoV‐2, assessing the efficacy of clinical treatment, population screening in the event of an outbreak, and epidemiological investigation.^16^, ^17^, ^18^ In response to a large number of oropharyngeal swabs samples, the detection efficiency of samples should be improved as soon as possible, reports should be issued in time, and suspected cases should be screened in a timely and rapid manner in the first place, so as to optimize patients' medical experience and effectively prevent and control the epidemic.^19^ Therefore, POCT, which can rapidly detect viral nucleic acid at the sampling site, has greatly enriched the viral nucleic acid detection scene and is an effective complement to SARS‐CoV‐2 laboratory nucleic acid detection.^20^, ^21^, ^22^

SARS‐CoV‐2 rapid testing reagent of Daan gene and its supporting equipment have been approved by the National Health Commission of China. From the statement provided by the manufacturers, as compared to the conventional nucleic acid detection system, the SARS‐CoV‐2 rapid nucleic acid detection system (Daan Gene) is equipped with a fluorescent quantitative PCR system with the characteristics of small size, light weight, easy operation, and fast speed (the amplification detection time only takes 30 min). In addition, the supporting reagents add an anti‐pollution system that can degrade the pollutants generated in the PCR process and reduce the false positives caused by the PCR product pollution, including UDG enzyme and dUTP enzyme; as well as a high‐efficiency enzyme system that can improve the specificity and sensitivity of PCR reaction, including hot start Taq antibody enzyme and c‐mmlv enzyme.^23^ However, so far relatively few studies have compared the diagnostic performance between this testing system and the SARS‐CoV‐2 conventional nucleic acid detection system.

Overall, results of this study showed that the sensitivity of the SARS‐CoV‐2 rapid nucleic acid detection system (Daan Gene) was slightly lower than that of the conventional detection system, but their specificity for SARS‐CoV‐2 was equivalent.

And the SARS‐CoV‐2 rapid nucleic acid detection system (Daan Gene) had ≥98.0% PPV and NPV for the detection of SARS‐CoV‐2. Moreover, the SARS‐CoV‐2 rapid nucleic acid detection system (Daan Gene) demonstrated an excellent test agreement with the widely used conventional nucleic acid detection system (κ = 0.893, *p* = 0.000). The diagnostic sensitivity of a nucleic acid test kit is closely related to the viral load of the sample.^24^ Discordant specimens in this study all had relatively high *C*_t_ values (indicative of a lower viral concentration in the specimen), ranged from 33.67 to 44.26, with median of 38.14, IQR of 2.95. Among the six different nucleic acid testing laboratories, the SARS‐CoV‐2 rapid nucleic acid detection system (Daan Gene) showed the lowest sensitivity and positive coincidence rate in HGP medical laboratory. The inconsistent results of molecular detection in this laboratory were also 14 samples with low concentration of viral RNA, whose Ct values ranging from 35.07 to 39.41, with median value of 37.55, and IQR of 2.71.

Additionally, according to one of the criteria for lifting quarantine management and discharging from hospital in *China's COVID‐19 diagnosis and Treatment Protocol (Trial Edition 9)*, the *C*_t_ values of N gene and ORF gene in two consecutive SARS‐CoV‐2 nucleic acid tests were all ≥35. We further divided the viral loads of samples by the Ct values and calculated the sensitivity of these two systems under different viral loads. Results showed that the sensitivity of SARS‐CoV‐2 rapid nucleic acid detection system (Daan Gene) was 99.31% for specimens with high viral load (*C*_t_ < 35) and 50% for low viral load (*C*_t_ ≥ 35). SARS‐CoV‐2 rapid nucleic acid detection system (Daan Gene) performed less well at specimens with *C*_t_ values ≥35; however, the reduction in sensitivity is relatively unimportant since high *C*_t_ values probably indicate a low transmission risk.^25^ Viral load tends to be higher at the beginning of infection, that is, when the virus is most infectious.^26^, ^27^, ^28^ Epidemiologically, high viral load carriers are more likely to be super‐spreaders. In a preliminary clinical study shown that patients with Ct values equal or above 34 do not excrete infectious viral particles and thus can be discharged from hospital care or strict confinement for non‐hospitalized patients.^29^ It is generally believed that the virus culture in samples with *C*_t_ > 30 is negative, and these infected individuals are non‐infectious.^30^, ^31^ Furthermore, the sensitivity displayed by SARS‐CoV‐2 rapid nucleic acid detection system (Daan Gene) for specimens with *C*_t_ values ≥35 was still substantially higher than that most of the SARS‐CoV‐2 rapid antigen tests. Michael Kleines et al concluded that the SARS‐CoV‐2 Rapid Antigen Test (Roche)'s sensitivity for samples with a *C*_t_ value of <25, 25–30, 30–35, and ≥35 was 100%, 95%, 44.8%, and 22.2%, respectively.^24^ Valeria Ghisetti et al indicated that the detection rate of the SARS‐CoV‐2 Rapid Antigen Test (SD‐Biosensor) was 100% for samples with a *C*_t_ < 28 and decreased to 38.5%, 26.7%, and 9.1% in the other ranks (*C*_t_ ≤ 25, 25–28, 28–30, 30–35, >35).^32^ Dominic N.C. Tsang et al shown that the Panbio COVID‐19 Ag Rapid Test Device showed 83.3%–100% sensitivity for both high viral load and normal viral load specimens (*C*_t_ ≤ 24.22), but 0%–11.1% sensitivity for low viral load specimens (*C*_t_ ≥ 31.43).^33^ Anaïs Scohy et al.^34^ reported that the overall poor sensitivity of the COVID‐19 Ag Respi‐Strip did not allow using it alone as the frontline testing for COVID‐19 diagnosis, with an overall sensitivity of 30.2%. Therefore, although the sensitivity of SARS‐CoV‐2 rapid nucleic acid detection system is not as high as that of SARS‐CoV‐2 conventional nucleic acid detection system, SARS‐CoV‐2 rapid nucleic acid detection system is a rapid and simple detection method that can identify COVID‐19 patients with high transmission risk and may be helpful to check the virus clearance rate of patients.

## 5 CONCLUSIONS

In summary, although the sensitivity of SARS‐CoV‐2 rapid nucleic acid detection system is not as high as that of SARS‐CoV‐2 conventional nucleic acid detection system, SARS‐CoV‐2 rapid nucleic acid detection system is a rapid and simple detection method that can identify COVID‐19 patients with high transmission risk. For emergency and symptomatic patients, or for large‐scale population screening at customs, railway stations, airports, etc., it is a better choice to use the SARS‐CoV‐2 rapid nucleic acid detection system for early triage and rapid management of the suspected population, and then use the conventional nucleic acid detection system for confirmation. It is believed that with the development of rapid nucleic acid detection system, more stable and high‐quality SARS‐CoV‐2 rapid nucleic acid detection technologies will emerge and become an indispensable force in clinical diagnosis and treatment and epidemic prevention and control.

## AUTHOR CONTRIBUTIONS

All authors have made substantial contributions to all of the following: Chan Mo and Xiuming Zhang, the conception and design of the study; Kamfai Lo, Ying He, and Bo Peng, acquisition of data; Feifan Guo, Zhou Zheng, and Ruiwei Jiang, analysis and interpretation of data; Chan Mo, Yihua Cai, Yumin Li, Dongyue Guo, and Bing Zhang, drafting the article; Tong Ou and Dan Xiong, revising the article critically for important intellectual content; Chan Mo, Dan Xiong, and Xiuming Zhang, final approval of the version to be submitted.

## FUNDING INFORMATION

This work was supported by Shenzhen Key Medical Discipline Construction Fund (Grant No. SZXK054), Science and Technology Planning Project of Shenzhen City of China (Grant No. JCYJ20190812171816857, JCYJ20180306172209668).

## CONFLICT OF INTEREST

The authors declare no conflict of interest.

## Data Availability

The datasets used and/or analysed during the current study are available from the corresponding author on reasonable request.
